# The Exploitation of Single-Chambered Microbial Fuel Cells for PET Removal in Water

**DOI:** 10.3390/microorganisms13112500

**Published:** 2025-10-31

**Authors:** Andre Hadji-Thomas, Shuyao Wang, Yvan Gariepy, Vijaya Raghavan

**Affiliations:** Bioresource Engineering, Faculty of Agricultural and Environmental Sciences, McGill University, 21111 Lakeshore Road, Sainte-Anne-de-Bellevue, QC H9X 3V9, Canada; andre.hadji-thomas@mail.mcgill.ca (A.H.-T.); yvan.gariepy@mcgill.ca (Y.G.); vijaya.raghavan@mcgill.ca (V.R.)

**Keywords:** microbial fuel cell, microplastic degradation, polyethylene terephthalate (PET) degradation

## Abstract

This work investigated the use of microbial fuel cells (MFCs) for the degradation of polyethylene terephthalate (PET) and the simultaneous generation of electricity. The study implemented two separate single-chamber MFCs, one with a co-culture of *Ideonella sakaiensis* and *Geobacter sulfurreducens* (I.S-G.S) and the other with *Ideonella sakaiensis* and activated sludge (I.S-AS). The effectiveness of microplastic (MP) degradation was assessed based on the electroactivity of the anodic biofilm, the reduction in particle size, and the decrease in PET mass. Both systems achieved a significant reduction in MP size and mass, with the I.S-AS system notably surpassing the I.S-G.S in terms of efficiency and electricity generation. The I.S-AS system achieved a 30% mass reduction and 80% size reduction, along with a peak voltage of 222 mV. The study concludes that MFCs, particularly with the activated sludge co-culture, offer a viable and more environmentally friendly alternative for MP degradation and energy recovery. These findings suggest a promising direction for improving waste management practices and advancing the capabilities of bio-electrochemical systems in addressing plastic pollution. Further research is recommended to optimize the operational conditions and to test a broader range of MP sizes for enhanced degradation efficacy.

## 1. Introduction

For the past 50 years, plastic use has grown exponentially. According to the United Nations Environment Program (UNEP), approximately one million plastic bottles are consumed every minute, and the global use of plastic bags reaches up to five trillion per day [1]. Plastic pollution is one of the greatest threats to humanity and the environment in the 21st century. Even though a portion of plastic consumption is recycled, a large share still ends up in natural ecosystems.

Microplastics (MPs) are plastic particles smaller than 5 mm. MPs can enter environmental systems via multiple pathways, including landfill disposal, release from wastewater treatment plants, application of treated sludge and compost in agriculture, atmospheric deposition, and the use of plastic mulches in farming practices [2]. The release of plastics into the environment has led to the widespread presence of MPs in almost all ecosystems, which UNEP identifies as a growing threat to many organisms [3]. Polyethylene terephthalate (PET) is particularly prominent due to its extensive use and wide range of applications [4]. PET is a synthetic polymer composed of ethylene glycol and terephthalic acid, providing notable mechanical strength and flexibility. PET’s molecular structure, characterized by repeating ester units, is responsible for its durability and resistance to biodegradation, contributing to its persistence in natural environments. PET particles bioaccumulate in organisms, including agricultural plants, where they can affect yield [5], soil fauna such as earthworms [6], and aquatic organisms [7]. It has been reported that micro and nano plastics, including PET, can cross the blood–brain barrier, and their presence in the brain may lead to neurological disorders in mammals and aquatic species [8]. Consumer disposal of PET products, such as bottles and packaging, is a significant pathway of PET release into natural environments, often due to inadequate recycling and waste management systems [9]. Additionally, the wear and tear of PET-containing products, like synthetic textiles, release microplastic fibers into water during washing, which subsequently enter rivers and oceans [10]. Industrial spillage and mishandling of PET pellets during manufacturing and transportation further increase environmental PET loads [11]. Notably, the degradation of larger PET items into MPs enables their spread into remote regions, exacerbating the impact on wildlife and ecosystems [12]. Addressing these sources requires a multifaceted approach, emphasizing enhanced waste management, increased consumer awareness, and innovative recycling technologies. Physical methods (photodegradation and thermal degradation) and chemical methods (such as hydrolysis, glycolysis, alcoholysis, and acid hydrolysis) can break down PET in water, but the former are often slow or produce harmful by-products, while the latter require high temperatures and harsh chemicals, leading to cost and safety concerns [13,14,15,16,17,18]. These limitations highlight the urgent need for more sustainable and efficient solutions [19].

Bio-electrochemical systems (BESs) and in particular microbial fuel cells (MFCs) present a great advantage in terms of organic matter removal in water. MFCs produce energy by harnessing the catalytic activities of microorganisms to degrade chemical bonds in organic compounds [20]. A diverse array of microorganisms has demonstrated the ability to degrade MPs [21]. Yet, within this group, electroactive bacteria have not been documented. Recent research from Cambridge University has shed light on a groundbreaking approach: *Ideonella sakaiensis* (*I. sakaiensis*), known for its PET-degrading abilities, can be co-cultured with *Geobacter sulfurreducens* (*G. sulfurreducens*) to degrade PET. This co-culture allows the degradation of PET while releasing electrons [22].

Under anaerobic conditions, *Ideonella sakaiensis* degrades PET plastic by secreting PETase, which converts PET into mono(2-hydroxyethyl)-terephthalic acid (MHET). MHET is then hydrolyzed into TPA and EG. EG undergoes dehydration to acetaldehyde, which is further transformed into ethanol and acetyl-CoA. Acetyl-CoA is converted into acetate with the generation of ATP through substrate-level phosphorylation. Both acetate and ethanol are excreted, while in a co-culture with *Geobacter sulfurreducens*, acetate serves as an electron donor for *G. sulfurreducens* to produce electricity via extracellular electron transfer, resulting in the conversion of PET plastic into electricity and the production of valuable chemicals like acetate and ethanol, offering a sustainable approach for plastic waste mitigation and energy production. It was also observed that once PET is fully degraded under anaerobic conditions, the ethanol produced as an intermediate is further transformed into acetate through a series of enzymatic reactions [22].

This study investigates how single-chamber microbial fuel cells (SC-MFCs) can degrade PET while generating electricity. By examining two SC-MFC systems, one hosting a co-culture of *I. Sakaiensis* and *G. sulfurreducens*, and another—tested for the first time—combining *I. Sakaiensis* with activated sludge, this research aims to quantify the degradation potential. Leveraging biofilm electroactivity, particle size reduction, and PET mass loss, the study aims to quantify the degradation potential of these systems and critically evaluate their efficiency relative to existing challenges posed by conventional MP removal methods.

## 2. Materials and Methods

### 2.1. Experimental Procedure

To evaluate the effectiveness of MP removal using MFCs, two separate single-chamber MFCs (SC-MFCs) were operated for 30 days, one containing a co-culture of *I. sakaiensis* and *G. sulfurreducens* (I.S-G.S), and the other containing *I. sakaiensis* and activated sludge (I.S-AS). The first phase of the experiment aimed to determine the potential for MP degradation by these co-cultures within the MFCs. Once degradation capability was confirmed, the second phase assessed the removal efficiency in order to compare the performance of the two co-culture systems.

Following the study by Kalathil et al. [22], which reported the breakdown of 100 mg of PET over 30 days in a bio-electrochemical system, the experimental time frame was set to 30 days to enable a direct comparative analysis. To ensure accurate quantification, 1 g of PET of size 7.5 mm^2^ was introduced into each SC-MFC system at the start of the experiment.

### 2.2. Design of Single-Chamber MFC

**Figure 1:**
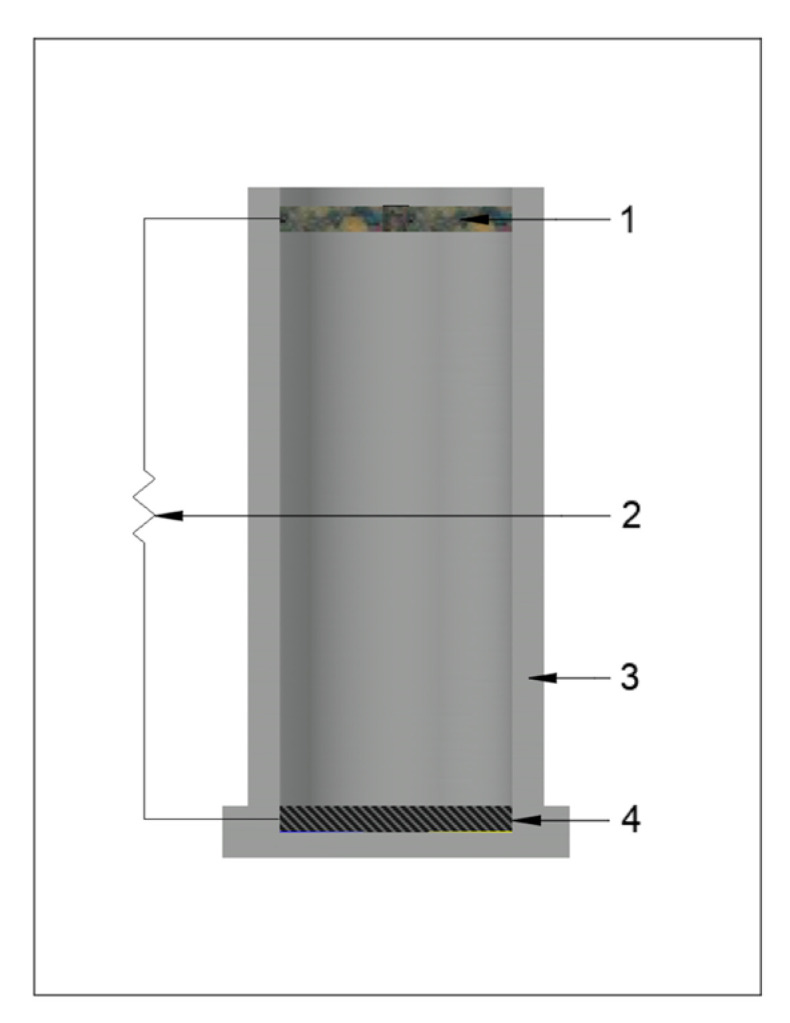
Schematic diagram of the single-chamber microbial fuel cell (SC-MFC), (1) air cathode; (2) external resistance; (3) PVC tube; (4) carbon felt anode.

In this experiment, polyvinyl chloride (PVC) water pipes were used to build single-chamber MFCs (Figure 1). A carbon felt anode was placed horizontally on the button, and a round floating air-cathode was placed on the surface of the solution. The air cathode is a 3D carbon sponge with carbon nano-particles, constructed based on Wang et al.’s method [23]. The distance between the anode and cathode was 150 mm. The insulated stainless-steel wires were used as the current collectors and connected with the electrodes by conductive cement.

In the two MFC setups, the solution is meticulously prepared to support the growth and activity of *I. sakaiensis* and the equivalent co-culture. The solution initially contains 1 g of PET, serving as the primary substrate for the bacteria. To ensure optimal growth conditions, two nutritive media, DSM 826 and activated sludge buffer, are supplemented in each setup, respectively, to provide essential nutrients and vitamins to the complement bacteria. Additionally, acetate is introduced into the solution at the outset. This initial addition of acetate is a strategic measure, intended to sustain the bacterial culture during the initial phase before the onset of PET degradation. This approach ensures a stable and efficient degradation process.

### 2.3. Polarization Test (P-Test)

**Figure 2:**
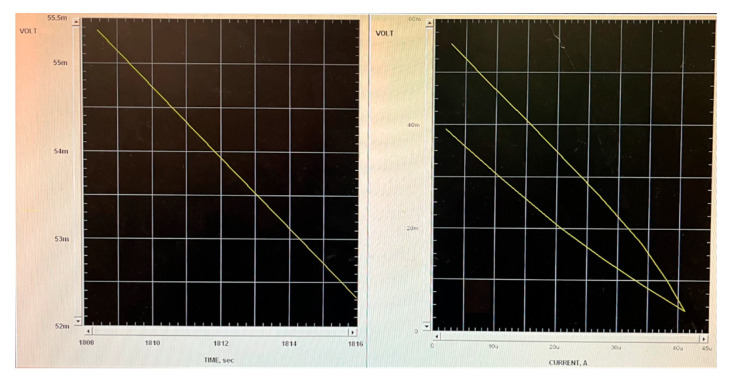
I.S-G.S Polarization Curve.

**Figure 3:**
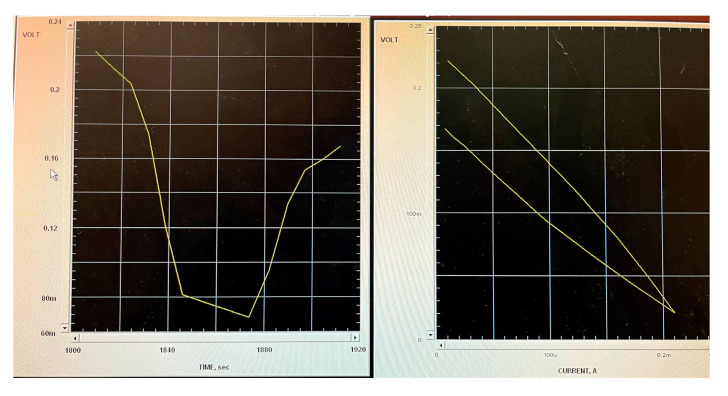
I.S-AS Polarization Curve.

The internal resistance (R_int_) was determined using a polarization test (P-test) as described by Mehta et al. [24]. This method was chosen because it allows for the precise characterization of the cell’s electrochemical behavior under varying loads, enabling the identification of the optimal operating resistance and the evaluation of internal losses. The process was as follows: initially, the open circuit voltage (OCV) was measured by disconnecting any external load for a period of 30 min. Afterward, the external load was reconnected, and its resistance was methodically reduced through 5 to 7 incremental steps. Each step was maintained for 10 min to ensure the voltage reached a stable condition. Voltage readings were taken at the conclusion of each interval. The procedure commenced with a high resistance setting and was concluded upon observing a significant decrease in current. These observations were then utilized to generate polarization (voltage vs. current) and power (output power vs. current) charts. From the polarization grap (Figure 2 and Figure 3) the portion that represented ohmic losses was linear and was used to calculate the total internal resistance (R_int_), based on the slope derived from linearly approximating the relevant data points [24]. The external resistance (R_ext_) was equal to the R_int_.

### 2.4. Bacterial Activation and Inoculation

The bacterial cultures were ordered from the Leibniz Institute DSMZ German Collection of Microorganisms and Cell Cultures GmbH (Braunschweig, Germany).The inoculation process was carried out in the recommended medium according to the following protocols.

#### 2.4.1. Inoculation of *I. sakaiensis*

To activate freeze-dried *I. sakaiensis*, sterile phosphate-buffered saline (PBS) was prepared, which was autoclaved at 121 °C for 15 min if sterility was uncertain. The freeze-dried bacteria were opened under sterile conditions and rehydrated by adding 1–5 mL of sterile PBS directly into the vial, mixing gently to ensure the bacteria were completely dissolved. R2A medium was prepared and autoclaved to sterilize, then poured into sterile Petri dishes once cooled to about 50 °C, allowing the agar to solidify at room temperature for 30–60 min, and subsequently dried overnight to reduce surface moisture. The solidified agar was inoculated with the rehydrated bacteria using sterile techniques to evenly spread the culture. The inoculated plates were incubated at 32 °C for 72 h under aerobic conditions. After incubation, the plates were examined for bacterial growth, and subcultures from well-isolated colonies were prepared for further analysis or propagation, with aseptic conditions maintained throughout the process to avoid contamination. All materials were disposed of in accordance with biosafety protocols [25].

#### 2.4.2. Inoculation of *G. sulfurreducens*

To prepare Medium 826 for *G. sulfurreducens*, the base ingredients, including NH_4_Cl, Na_2_HPO_4_, KCl, and Na-acetate, were combined in distilled water. Before adding NaHCO_3_, Na_2_-fumarate, and vitamins, the solution was sparged to create an anoxic environment. Then, NaHCO_3_ was added, and the pH was adjusted to 6.8 under the same gaseous conditions. The medium was dispensed into anoxic serum vials, sealed, and autoclaved. After autoclaving, sterile-filtered fumarate and vitamins were added, and the pH was re-adjusted if necessary [26].

For inoculating *G. sulfurreducens*, sterile anaerobic Medium 826 was used. The culture of *G. sulfurreducens*, typically stored in freezer stocks with DMSO, was thawed and inoculated into the prepared medium under anaerobic conditions. The bacteria were cultivated in this medium at 30 °C to reach mid-exponential growth phase before transferring to MFCs for further studies [4,27,28].

#### 2.4.3. Inoculation of Activated Sludge

Activated sludge previously maintained in a liquid medium at a temperature of 4 °C was mixed with an equivalent volume of buffer solution utilizing a vial shaker apparatus. Subsequently, this composite mixture was then poured onto the anode interface to initiate colonization as a preparatory step for the commencement of the experiment.

### 2.5. Measurement Methods

#### 2.5.1. Electroactivity Measurements

The electroactivity of the anodic biofilm was measured using a voltage monitoring system across the external resistor. Voltage was recorded using the Agilent 34970A Data Acquisition/Switch (Agilent Technologies, Santa Clara, CA, USA).

#### 2.5.2. Physiological Measurements

At the conclusion of the MFC process, the solution was filtered using a 20 μm sieve to retain MPs. Both the MFC solution and electrodes were rinsed above the sieve to capture any additional MPs that had adhered. The retained particles were collected and dried at low temperature to remove moisture without altering their structure. The dried particles were weighed, and a random sampling of 20 particles from each group was analyzed for surface area. A gram of unprocessed, ground PET was subjected to the same procedure to establish a 2% weight loss correction factor. Particle size was quantified using the same sampling method.

## 3. Results and Discussion

Upon concluding the experimental protocol, several metrics were measured to assess the experiment’s efficacy. These metrics included the mass reduction in MPs, serving as an indicator of biodegradation by *I. sakaiensis*; the reduction in PET particle size, denoting the degradative processes facilitated by *I. sakaiensis*; and electroactivity measurements, providing insights into the metabolic functions of the co-cultured bacterial species, either *G. sulfurreducens* or activated sludge.

### 3.1. Mass Reduction

Upon the experiment’s completion, the MP particles were collected in accordance with the methodology outlined in the methods section. Table 1 presents the quantitative data illustrating the mass reduction from the initial 1 g of PET. The concluding dry weight of MPs post-treatment with activated sludge and *G. sulfurreducens* was documented at 700 mg and 800 mg, respectively. This is in contrast to the pretreated benchmark of 1000 mg. Consequently, the aggregate weight loss of MPs was determined to be 30% for samples treated with activated sludge and 20% for those exposed to *G. sulfurreducens*.

### 3.2. Size Reduction

**Figure 4:**
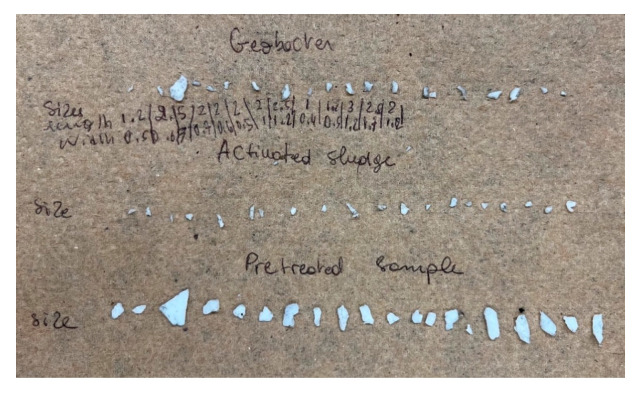
The changes of MP particles before after treatment.

Table 2 shows the results of MPs particle size reduction across different MFC treatments. As previously described, a subset of twenty particles was selected from each treatment group to evaluate the diminution in the surface area of PET particles. Figure 4 shows the segregated samples according to their respective treatment categories. The untreated particles, which did not undergo MFC-mediated degradation, are shown at the base of the image. Centrally positioned were particles subjected to the I.S-AS treatment, while the upper segment displays particles treated by the I.S-G.S process. Quantitative analysis of size (mm^2^) revealed a residual dimension of 7.5 mm^2^ for the control group, a reduced measure of 1.48 mm^2^ post I.S-AS treatment, and 3.49 mm^2^ following the I.S-G.S treatment. 

### 3.3. Electroactivity

**Figure 5:**
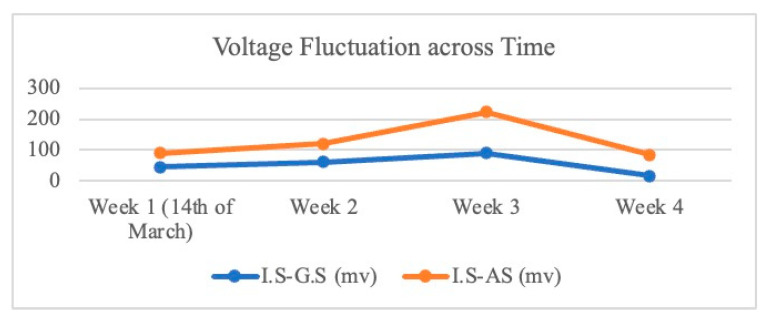
Voltage fluctuations of I.S-G.S and I.S-AS co-cultures over time.

Figure 5 illustrates the evolution of average voltage over time for each MFC. In the first week, the MFC voltages began at 46 mV for I.S-G.S and 90 mV for I.S-AS. By the second week, these values had increased to an average of 62 mV for I.S-G.S and 120 mV for I.S-AS. The upward trend continued until a peak was reached at 90 mV for I.S-G.S and a notable 222 mV for I.S-AS. After reaching these peak values, a P-test was conducted. To conclude the experiment, the circuit was modified by introducing a R_ext_, which mirrored the R_int_ values of 1200 Ω for I.S-G.S and 2200 Ω for I.S-AS, thereby effectively closing the circuit. The maximum current, power, and power density produced by I.S–G.S were 0.075 mA, 0.0068 mW, and 85.99 mW/m^3^, respectively, while those of I.S–AS were 0.1 mA, 0.022 mW, and 280.26 mW/m^3^. In addition, although the I.S-G.S system exhibited a slightly lower R_int_ compared to I.S-AS, the I.S-AS system demonstrated higher power output and stronger electrochemical activity. This suggests that the electron transfer efficiency in I.S-AS was influenced not only by R_int_ but also by the biocatalytic performance and conductive properties of the anodic biofilm. As reported by Rabaey et al. [29], the R_int_ is primarily determined by anode resistance, cathode resistance, electrolyte resistance, and membrane resistance. Anode resistance is mainly decided by the size of the anode and the activity of the exoelectrogenic bacteria, as well as both the anode biofilm and planktonic bacteria in bulk solution, which contribute to the power production of MFCs in the mixed bacterial culture environment [29]. In our setup, the anode, cathode, and nutrient medium were identical, and no membrane was used. Therefore, differences in R_int_ mainly reflect variations in the anode-associated processes, particularly the structure and activity of the biofilm. The slightly higher R_int_ observed in I.S-AS may result from a denser and more compact biofilm, which, while increasing resistance to ion diffusion, enhances cell–electrode connectivity and promotes more effective extracellular electron transfer (EET). As a result, despite its marginally higher R_int_, the I.S-AS system achieved superior overall electron transfer efficiency and electrochemical performance compared to the I.S-G.S system.

### 3.4. Discussion

This study examined the co-culture of *I. sakaiensis* with *G. sulfurreducens* and activated sludge in MFCs for the removal of MPs and the simultaneous production of electricity. The results demonstrated the practical viability of these co-cultures, especially when using activated sludge, in effectively degrading MPs. Notably, this research was the first documented instance of *I. sakaiensis* being utilized alongside activated sludge for MP degradation. A significant reduction in MP size was observed, coupled with measurable electricity generation, which confirmed the degradation capabilities of *I. sakaiensis* and the electricity-producing potential of the co-culture systems in an SC-MFC.

#### 3.4.1. Result Analysis

The I.S-G.S co-culture relied on the unique metabolic attributes of both bacteria. *I. sakaiensis*, known for its PET-degrading enzyme PETase, acted in synergy with *G. sulfurreducens*, a bacterium proficient in transferring electrons to anodes. Our data indicated that this co-culture produced a moderate rate of MP degradation, with a 20% reduction in mass and a 54% reduction in particle size. While significant, this system’s electricity generation, peaking at 90 mV, did not reach the optimal levels anticipated for MFCs [30]. This shortfall may have reflected the need for improved synchronization between the PET degradation pathway of *I. sakaiensis* and the electron transfer capabilities of *G. sulfurreducens*. The potential for fine-tuning this co-culture lies in optimizing conditions, such as nutrient availability, to facilitate PETase activity and electron transport, thereby compensating for the natural instability of co-cultures. In this study, the I.S-G.S co-culture degraded 200 mg of PET over 30 days, averaging 40 mg every 6 days. This degradation rate was significantly higher than the 23 mg reported over the same timeframe by Kalathil et al. [22]. This finding highlighted the enhanced efficiency of utilizing a co-culture within an SC-MFC for PET degradation.

*I. sakaiensis* initiates PET degradation through the secretion of PETase and MHETase, hydrolyzing PET into ethylene glycol (EG) and terephthalic acid (TPA) [31]. These intermediates can serve as carbon and electron sources for other electroactive microorganisms in the co-culture. In the I.S-G.S system, *G. sulfurreducens* oxidizes these metabolites and transfers electrons to the anode via conductive pili and c-type cytochromes, leading to current generation. In the I.S-AS system, the microbial community within activated sludge enhances this process by providing a more diverse set of metabolic pathways and electron shuttles, improving both degradation and electricity output. The observed differences between the two systems can thus be attributed to their distinct microbial electron transfer efficiencies and metabolic compatibility. While PET degradation primarily depends on enzymatic hydrolysis by *I. sakaiensis*, electricity generation relies on the capacity of associated microorganisms to oxidize degradation intermediates and transfer electrons to the electrode. The stronger performance of the I.S-AS system suggests that the complex microbial community facilitated more efficient substrate utilization and electron flow.

In contrast, the I.S-AS co-culture showed improved efficiency in both MP degradation and electricity production. A 30% mass reduction and an 80% reduction in particle size demonstrated the potential of this co-culture. Additionally, the peak voltage reached 222 mV, indicating robust electrogenic activity. This enhanced performance may have been due to the diverse microbial community present in activated sludge, which formed a more robust and adaptable biofilm. The superior performance of the I.S-AS co-culture could also be linked to its resilience and adaptability to environmental changes, a characteristic of activated sludge communities. Furthermore, activated sludge was better adapted to the semi-aerobic medium of the SC-MFC environment than the strictly anaerobic *G. sulfurreducens*. This adaptability rendered the I.S-AS system a more versatile and potentially impactful bioremediation strategy.

The R_int_, as measured through polarization curves, suggested a more efficient electron transfer in the I.S-AS system compared with the I.S-G.S system. This efficient electron flow was essential for maximizing power output and is a desirable trait in bio-electrochemical systems aimed at energy recovery from waste. It is important to note that in real-world scenarios, MPs often exist in much smaller sizes than the 5 mm particles used in this study, which would present a greater surface area for enzymatic action. Thus, for both systems, utilizing a range of MP sizes that mimic environmental conditions may not only enhance biodegradation rates but also provide a more accurate assessment of real-world applicability.

#### 3.4.2. Limitation and Future Perspective

The study introduced a novel approach to MP degradation and electricity generation, yet it acknowledged areas for improvement. Although the electricity outputs were encouraging, they remained below the benchmark range of 300–500 mV typical for MFCs [30]. This shortfall underscored a potential inefficiency in the co-culture systems’ metabolic processes, suggesting that the current operational conditions may not fully harness the electrogenic potential of the cultures. Moreover, the use of larger MPs, nearing the upper size limit of 5 mm, may have restricted the accessibility of the bacterial enzymes to the plastic substrates, potentially limiting *I. sakaiensis*’s degradative efficiency. This observation emphasized the critical influence of particle size on the degradation rate and underscores the necessity to replicate environmental conditions more closely in future experiments.

Despite these limitations, the research provided a substantial foundation for the development of bio-electrochemical systems in MP degradation. It demonstrated the feasibility of using MFCs as a sustainable solution to plastic pollution and highlighted the prospect of integrating waste treatment with energy recovery, reinforcing the concept of a circular economy. The applicability of these results was broad, marking a significant step toward non-destructive, cost-effective, and environmentally friendly solutions for waste management, particularly in wastewater treatment contexts. Furthermore, these findings suggested promise for enhancing circular economy models by converting waste into valuable energy resources.

To advance the field, there is a compelling need to conduct a more detailed evaluation of the nutritional profiles and environmental parameters influencing MFC performance. Optimizing these factors could lead to improved electrical outputs and a deeper understanding of microbial interactions within co-cultures. An immediate priority for future research should be to test a wider range of microplastic sizes, with particular emphasis on smaller particles that are more representative of environmental conditions. This will help determine whether these finer microplastics enhance microbe–substrate interactions and consequently improve degradation rates. To extend the practical applications of MFCs in environmental remediation, future studies should also examine the dynamics of MFCs with varying concentrations of MPs, reflecting the lower levels typically found in natural water systems [32]. Furthermore, it would be advantageous to regularly monitor microbial interactions by systematically collecting samples for bacterial activity and chemical analysis during the experiment. This methodical approach would help in developing a comprehensive understanding of the anodic reactions and interactions within microbial fuel cells. These directions would not only increase the relevance of the research findings but also propel this technology toward larger-scale operations that can be used in wastewater treatment plants.

## 4. Conclusions

This work investigated the use of SC-MFCs for degrading microplastics, specifically PET, while also generating electricity. The research introduced a novel co-culture of *I. sakaiensis* with activated sludge in an SC-MFC, marking the first application of such a system. This co-culture demonstrated enhanced PET degradation and energy generation, suggesting its potential utility in sustainable waste management practices.

Empirical results indicated a substantial decrease in both the size and weight of PET particles, signifying an effective degradation pathway within the co-culture. Particularly notable was the activated sludge system, which produced higher-than-anticipated levels of electrical output. These findings reinforced the dual advantage of the SC-MFCs, providing a method to address plastic pollution and generate energy concurrently.

The study helped to bridge the gap between the escalating problem of plastic pollution and the search for effective biotechnological solutions. Referencing initial environmental concerns, such as those from the UNEP, the thesis highlighted the urgency and applicability of the research findings. It presented a method that simultaneously addresses environmental degradation and resource recovery, aligning with broader sustainability objectives.

Future research should focus on optimizing the operational variables of SC-MFCs and studying the interactions between diverse microbial populations and microplastics of various concentrations and dimensions. By deepening the understanding of these systems, improvements in degradation efficiency and scalability of the technology could be achieved, leading to broader applications in environmental management and clean energy production. Implications for practical deployment are significant, suggesting the possibility of transforming waste treatment processes by integrating such technologies to convert MPs into electricity, which are usually untreated and released into natural ecosystems. This work provides a foundation for further exploration and serves as a prompt for those in related fields to consider the practical implications of these findings.

## Figures and Tables

**Table 1:** Comparative Results of MPs Mass Reduction in Different MFC Treatments.

Characteristics Measured	Activated Sludge	*G. sulfurreducenns*	Pretreated
MP dry weight (mg)	700	800	1000
MP total weight loss (mg)	300	200	/
Total weight loss (%)	30	20	/

**Table 2:** Results of MPs Particle Size Reduction Across Different MFC Treatments.

Experimental Results	I.S-AS	I.S-G.S	Untreated
MP Particle Size (mm^2^)	1.48	3.49	7.5
MP Particle Size Loss (%)	80	54	/

## Data Availability

The original contributions presented in this study are included in the article. Further inquiries can be directed to the corresponding author.

## Abbreviations

The following abbreviations are used in this manuscript:

MFC	Microbial fuel cell
PET	Polyethylene terephthalate
MP	Microplastic
UNEP	United Nations Environment Program
BES	Bio-electrochemical systems
*I. Sakaiensis*	Ideonella Sakaiensis
*G. sulfurreducens*	*Geobacter sulfurreducens*
SC-MFCs	Single-chamber microbial fuel cells
I.S-G.S	Co-culture of *I. sakaiensis* and *G. sulfurreducens*
I.S-AS	Co-culture of *I. sakaiensis* and activated sludge
PVC	Polyvinyl chloride
P-test	Polarization test
R_ext_	External resistance
OCV	Open circuit voltage
R_int_	Internal resistance
PBS	Phosphate-buffered saline
